# Antioxidant Activities and Lipid Accumulation-Inhibitory Effects of Seed and Callus Extracts of *Impatiens balsamina* L.

**DOI:** 10.3390/plants15111716

**Published:** 2026-06-01

**Authors:** Ye-Eun Ha, Ga-Ram Yu, Hyuck Kim, Dong-Woo Lim, Jai-Eun Kim

**Affiliations:** 1 Department of Life Sciences, Dongguk University, Gyeongju 38066, Republic of Korea; hkdp94@gmail.com; 2 Institute of Korean Medicine, Dongguk University, Goyang 10326, Republic of Korea; kalama2@dongguk.edu; 3 TOPO Lab., Co., Ltd., Goyang 10326, Republic of Korea; hk_ceo@topo.co.kr; 4 Department of Pathology, College of Korean Medicine, Dongguk University, Goyang 10326, Republic of Korea

**Keywords:** *Impatiens balsamina* L., seed extract, callus, antioxidant activity, flavonoids, lipid peroxidation, in silico network analysis

## Abstract

The seeds of *Impatiens balsamina* L. have been traditionally used in East Asian medicine and are known to contain bioactive compounds with antioxidant properties. However, studies focusing on seed-derived callus remain limited. This study aimed to comparatively evaluate the antioxidant activities and lipid accumulation-inhibitory effects of 70% ethanol extracts from seeds (IB) and seed-derived callus (IBC) of *I. balsamina*. Callus was induced on Murashige and Skoog (MS) medium supplemented with 2,4-dichlorophenoxyacetic acid (2,4-D). Antioxidant activities were evaluated using DPPH radical scavenging, superoxide anion scavenging, deoxyribose-based hydroxyl radical scavenging, DNA nicking, lipid peroxidation, and relative electrophoretic mobility (REM) assays, along with the determination of total phenolic, flavonoid, and tannin contents. Cell viability and lipid accumulation were assessed in FFA-treated HepG2 cells. In silico network and transcription factor (TF) enrichment analyses were performed to explore underlying mechanisms. Callus induction was most effective at 1 mg/L 2,4-D. Both IB and IBC exhibited antioxidant activities across all assays, with IB showing higher activity and greater phytochemical content than IBC. Both extracts reduced lipid accumulation in FFA-treated HepG2 cells at non-cytotoxic concentrations. Network analysis identified enrichment in pathways related to oxidative stress, inflammation, and lipid metabolism, and TF enrichment analysis identified NFKB1 and ATF3 as major upstream regulators. Both IB and IBC exhibited antioxidant activities across multiple in vitro assays, with IB showing higher activity attributable to its more complex phytochemical content. The lipid accumulation-inhibitory effects observed in FFA-treated HepG2 cells suggest a potential association between antioxidant capacity and lipid regulation, although the underlying mechanisms remain to be experimentally validated. Seed-derived callus may serve as a useful in vitro model for studying plant-derived bioactive compounds, pending further optimization.

## 1. Introduction

*Impatiens balsamina* L. is an annual herb belonging to the family Balsaminaceae [1]. Although it is native to South Asia, it is widely cultivated as an ornamental and medicinal plant across Asia and has been introduced to other regions. Ethnopharmacological studies have reported its traditional use in the treatment of rheumatism, pain, bruising, and nail inflammation, while the seeds have also been used to alleviate puerperal pain [2,3].

Phytochemical studies have shown that *I. balsamina* contains a variety of bioactive compounds, including phenolics, flavonoids, and naphthoquinones [4]. Extracts from the aerial parts, particularly stems and leaves, have demonstrated significant antioxidant activity, which is largely attributed to these phenolic and flavonoid constituents [5,6]. However, despite these findings, studies specifically addressing the antioxidant potential of the seeds remain limited, and information regarding seed-derived callus is scarce.

Seeds function as storage organs that accumulate specialized metabolites involved in protection during dormancy and germination, thereby contributing to cellular redox balance. In particular, phenolics and flavonoids are recognized as major non-enzymatic antioxidants that protect biomolecules from oxidative damage [7,8,9].

In contrast, callus tissues consist of dedifferentiated and unorganized cells generated under in vitro culture conditions and often exhibit reduced accumulation of secondary metabolites due to the incomplete development of tissue-specific biosynthetic pathways [10,11]. Therefore, comparative analysis of seeds and seed-derived callus provides a useful model for examining tissue-dependent differences in phytochemical composition and antioxidant activity [12].

Reactive oxygen species (ROS), including superoxide anions, hydroxyl radicals, and hydrogen peroxide, are continuously generated during normal metabolism. Although ROS play essential roles in cellular signaling, their excessive accumulation disrupts redox homeostasis and induces oxidative stress, leading to damage to lipids, proteins, and nucleic acids [13,14,15]. Oxidative stress is closely associated with the development and progression of various metabolic and degenerative diseases, highlighting the importance of identifying natural antioxidants capable of scavenging ROS [16].

In particular, oxidative stress plays a critical role in the development of lipid metabolic disorders, including hepatic steatosis, by promoting lipid accumulation and impairing lipid homeostasis [17]. FFA-induced lipid accumulation in hepatocytes, such as HepG2 cells, is widely used as an in vitro model to investigate oxidative stress-related lipid metabolism [18,19]. In this context, antioxidant compounds may contribute to the reduction in lipid accumulation by mitigating oxidative stress.

From a biotechnological perspective, plant callus cultures offer several advantages over whole-plant systems, including continuous production under controlled conditions independent of environmental and seasonal variations, and amenability to enhancement strategies such as elicitor treatment and bioreactor-based scale-up. Despite this potential, the antioxidant properties of seed-derived callus of *I. balsamina* and its relationship to the phytochemical composition of the corresponding seed extract remain largely unexplored, providing an additional motivation for the present study. In the present study, we comparatively evaluated the antioxidant activities of 70% ethanol extracts from the seeds (IB) and seed-derived callus (IBC) of *I. balsamina* to investigate tissue-dependent differences. Multiple in vitro assays were employed to assess radical scavenging activity, protection against oxidative DNA damage, and inhibition of lipid oxidation. In addition, the effects of IB and IBC on lipid accumulation were examined in FFA-treated HepG2 cells to explore the potential relationship between antioxidant activity and lipid regulation. Furthermore, a bioinformatics-based network analysis was employed to explore the key targets and associated pathways underlying these biological effects.

## 2. Materials and Methods

### 2.1. Reagents

Seeds were purchased from a commercial supplier (Cheongnong Seed Co., Bucheon, Republic of Korea) with taxonomic identification provided by the manufacturer based on standard commercial product specifications. As the seeds were obtained from a registered agricultural seed company subject to regulatory quality standards, formal herbarium voucher deposition was not applicable in this case. Murashige and Skoog (MS) basal medium, sucrose, agar, and plant growth regulators, including 2,4-dichlorophenoxyacetic acid (2,4-D), were obtained from Duchefa Biochemie (Haarlem, The Netherlands). For antioxidant assays, 2,2-diphenyl-1-picrylhydrazyl (DPPH), nitroblue tetrazolium (NBT), deoxyribose, thiobarbituric acid (TBA), trichloroacetic acid (TCA), and other analytical-grade reagents were purchased from Sigma-Aldrich (St. Louis, MO, USA), unless otherwise specified. Supercoiled pBR322 plasmid DNA used for the DNA nicking assay was obtained from Thermo Fisher Scientific (Waltham, MA, USA). Low-density lipoprotein (LDL) used for the relative electrophoretic mobility (REM) assay was purchased from Merck (Darmstadt, Germany). Copper sulfate (CuSO_4_) used for LDL oxidation was also obtained from Sigma-Aldrich. All solvents, including ethanol and methanol, were of analytical grade and obtained from commercial suppliers.

### 2.2. Plant Tissue Culture and Callus Induction

Seeds of *I. balsamina* were washed under running tap water and subjected to sonication (three times for 5 min each) for surface cleaning. The seeds were then surface-sterilized by immersion in 70% ethanol for 1 min, followed by treatment with 3% sodium hypochlorite (NaOCl) for 10 min. After sterilization, the seeds were rinsed three times with sterile distilled water (10 min each). An additional sterilization step using 5% NaOCl for 1 min was performed to ensure complete surface sterilization, followed by three further rinses with sterile distilled water (10 min each). The seeds were then air-dried under aseptic conditions in a laminar flow hood and inoculated onto a modified Murashige and Skoog (MS) medium consisting of MS basal salts and MS vitamins, supplemented with 30 g/L sucrose, 4 g/L Phytagel, and 1 g/L casein hydrolysate (see Table 1 for full composition), and further supplemented with 2,4-dichlorophenoxyacetic acid (2,4-D) at concentrations of 0.5, 1.0, 2.0, and 5.0 mg/L [20]. Twenty-five explants were cultured per plate for each treatment concentration. The pH of the medium was adjusted to 5.7 prior to autoclaving. Cultures were incubated in the dark at 28 ± 1 °C for 3 months to induce callus formation. Callus induction frequency (%) was calculated as the number of explants showing visible callus formation divided by the total number of explants cultured per treatment, multiplied by 100, and was recorded after 3 months of culture. Callus morphology was qualitatively evaluated based on visual inspection of callus size, compactness, surface appearance, color, and degree of browning. A three-level scoring system was applied: +, small and loosely organized callus with slight browning; ++, moderate callus formation with partial browning; +++, well-developed, compact, and glossy callus without browning. The morphological assessment was performed at the same time point as the frequency recording (3 months of culture).

### 2.3. Sample Extraction and Preparation

Dried seeds (65 g) and freeze-dried seed-derived callus (100.46 g) of *I. balsamina* were pulverized and extracted with 1 L of 70% ethanol under continuous stirring for 3 days at room temperature. The callus was harvested, washed with distilled water, and freeze-dried prior to extraction. Extract yields were calculated on a dry-weight basis. The extracts were filtered through Whatman No. 1 filter paper, and the filtrates were collected and concentrated under reduced pressure using a rotary evaporator to remove ethanol. The concentrates were re-dissolved in distilled water and partitioned with hexane (1:1, *v*/*v*) to remove non-polar constituents such as lipids and pigments, thereby enriching the aqueous fraction in polar phenolic and flavonoid compounds, which are the primary contributors to the antioxidant activities evaluated in this study. The aqueous layer was collected, concentrated under reduced pressure, and subsequently freeze-dried to obtain powdered extracts of seed (IB, 2.6 g, yield: 4.0%) and callus (IBC, 4.26 g, yield: 4.24%). The dried extracts were stored at −20 °C until further use.

### 2.4. DPPH Radical Scavenging Activity

The free radical scavenging activity of IB and IBC was evaluated using the 2,2-diphenyl-1-picrylhydrazyl (DPPH) assay according to the method of Brand-Williams et al., 1995 [21] with minor modifications. Briefly, 50 μL of sample solutions at various concentrations was mixed with 1 mL of 0.1 mM DPPH solution in ethanol and 450 μL of 50 mM Tris-HCl buffer (pH 7.4). The reaction mixture was incubated at room temperature in the dark for 40 min. After incubation, the absorbance was measured at 517 nm using a spectrophotometer. L-ascorbic acid was used as a positive control. DPPH scavenging activity (%) was calculated as follows: [(A_control − A_sample)/A_control] × 100. All experiments were performed in triplicate.

### 2.5. Superoxide Anion Scavenging Activity

The superoxide anion (O_2_•^−^) scavenging activity of IB and IBC was evaluated using a modified method of Gotoh and Niki. The reaction mixture consisted of 100 μL of 30 mM EDTA (pH 7.4), 10 μL of 30 mM hypoxanthine in 50 mM NaOH, and 200 μL of 1.42 mM nitroblue tetrazolium (NBT). IB and IBC at various concentrations were added to the reaction mixture. The mixture was pre-incubated at room temperature for 3 min, and the reaction was initiated by adding 100 μL of xanthine oxidase (0.5 U per reaction). The final reaction volume was adjusted to 3 mL with 50 mM phosphate buffer (pH 7.4). After incubation at room temperature for 20 min, the absorbance was measured at 560 nm using a spectrophotometer [22]. All experiments were performed in triplicate.

### 2.6. Deoxyribose Assay

The hydroxyl radical scavenging activity of IB and IBC was evaluated using the deoxyribose assay according to the method of Halliwell et al., with minor modifications [23]. Both non-site-specific and site-specific assays were performed to distinguish direct hydroxyl radical scavenging from metal ion chelation effects. For the non-site-specific assay, IB and IBC at various concentrations were added to a reaction mixture (1 mL) containing 100 μM FeCl_3_, approximately 100 μM EDTA, 1.5 mM H_2_O_2_, 2.5 mM deoxyribose, and 100 μM L-ascorbic acid in 50 mM phosphate buffer (pH 7.4). The reaction mixture was incubated at 37 °C for 1 h. After incubation, 1 mL of 0.5% (*w*/*v*) thiobarbituric acid (TBA) in 0.025 M NaOH and 1 mL of 2.8% (*w*/*v*) trichloroacetic acid (TCA) were added. The samples were heated at 80 °C for 30 min, cooled on ice, and the absorbance was measured at 532 nm. For the site-specific assay, EDTA was omitted from the reaction mixture to evaluate the contribution of metal ion chelation to hydroxyl radical inhibition by IB and IBC. All experiments were performed in triplicate.

### 2.7. DNA Nicking Assay

The protective effects of IB and IBC against oxidative DNA damage were evaluated using a DNA nicking assay based on Fenton’s reaction [24]. Supercoiled pBR322 plasmid DNA (1 μg) was treated with various concentrations of IB and IBC in the presence of Fenton’s reagents consisting of 30 mM H_2_O_2_, 0.05 mM ascorbic acid, and 80 μM FeCl_3_ in 10 mM phosphate buffer (pH 7.4), with a final reaction volume of 20 μL. The reaction mixtures were incubated at 37 °C for 15 min to induce oxidative DNA strand breakage. The reaction products were analyzed by electrophoresis on a 1% agarose gel in TAE buffer at 100 V for 30 min, followed by staining with ethidium bromide. The concentrations of IB (5 mg/mL) and IBC (20 mg/mL) used in this assay reflect the difference in antioxidant potency between the two extracts, as IBC consistently exhibited weaker activity than IB across all antioxidant assays. These concentrations were selected to allow observable protective effects to be demonstrated for both extracts within a single comparative experiment. The gel-based visualization of supercoiled (Form I), nicked circular (Form II), and linear (Form III) DNA forms is an internationally established qualitative criterion for assessing oxidative DNA strand breakage, and the distinct pattern of DNA form conversion observed in this study provides sufficient information to evaluate the comparative protective effects of IB and IBC.

### 2.8. Relative Electrophoretic Mobility (REM) Assay

The protective effects of IB and IBC against oxidative modification of low-density lipoprotein (LDL) were evaluated using a relative electrophoretic mobility (REM) assay [25]. Human LDL (120 μg/mL) was dissolved in 1× phosphate-buffered saline (PBS, pH 7.4) and pre-incubated with various concentrations of IB and IBC. Oxidation was initiated by the addition of 10 μM CuSO_4_, and the reaction mixtures were incubated at 37 °C for 12 h. After incubation, the samples were subjected to electrophoresis on a 0.7% agarose gel in TAE buffer at 85 V for 1 h. The gels were stained with Coomassie Brilliant Blue and destained with distilled water. The relative electrophoretic mobility of LDL was determined to evaluate the extent of oxidative modification. All experiments were performed in triplicate.

### 2.9. Lipid Peroxidation Inhibitory Activity

The inhibitory effects of IB and IBC on lipid peroxidation were evaluated using a modified thiocyanate method as described by Ohyashiki et al. [26]. Briefly, 0.4 mL of sample solution (final concentration: 0.01–3 mg/mL) was mixed with 0.2 mL of linoleic acid (25 mg/mL in 99% ethanol) and 0.4 mL of 50 mM phosphate buffer (pH 7.4). The reaction mixture was incubated at 40 °C for 15 min to allow lipid peroxidation to occur. After incubation, 0.1 mL of the reaction mixture was transferred to a new tube and mixed with 3 mL of 70% ethanol, 0.1 mL of ammonium thiocyanate solution (300 mg/mL, 30% *w*/*v*), and 0.1 mL of ferrous chloride solution (2.45 mg/mL FeCl_2_ in 3.5% HCl). The mixture was allowed to react for 3 min at room temperature. The absorbance was measured at 500 nm using a spectrophotometer.

All experiments were performed in triplicate.

### 2.10. Determination of Total Phenolic, Flavonoid, and Tannin Contents

Total phenolic content (TPC) was determined using the Folin–Ciocalteu method with gallic acid as the standard [27]. Briefly, 50 μL of IB and IBC solutions prepared in distilled water was mixed with 50 μL of 50% Folin–Ciocalteu reagent. After 3 min, 1 mL of 2% Na_2_CO_3_ solution was added, and the mixture was incubated at room temperature for 30 min. The absorbance was measured at 750 nm, and the results were expressed as mg gallic acid equivalents (GAE)/g extract. Total flavonoid content (TFC) was measured using the aluminum chloride colorimetric method with (+)-catechin as the standard [28]. Briefly, 250 μL of sample was mixed with 1 mL of distilled water and 75 μL of 5% NaNO_2_ solution. After 5 min, 300 μL of 10% AlCl_3_ solution was added. The mixture was allowed to react for 6 min, followed by the addition of 500 μL of 1 M NaOH. The absorbance was measured at 510 nm, and the results were expressed as mg catechin equivalents (CE)/g extract. Total tannin content (TTC) was determined using the Folin–Denis method with tannic acid as the standard [29]. Briefly, 100 μL of sample was diluted with distilled water to 7.5 mL, followed by the addition of 0.5 mL of Folin–Denis reagent and 1 mL of 20% Na_2_CO_3_. The volume was adjusted to 10 mL with distilled water, and the mixture was incubated at room temperature for 30 min. The absorbance was measured at 700 nm, and the results were expressed as mg tannic acid equivalents (TAE)/g extract. All experiments were performed in triplicate.

### 2.11. Cell Culture and Cell Viability Assay

HepG2 cells were cultured in Dulbecco’s modified Eagle medium (DMEM) supplemented with 10% fetal bovine serum (FBS) and 1% penicillin–streptomycin at 37 °C in a humidified incubator containing 5% CO_2_. To evaluate the effects of IB and IBC on cell viability, an MTT assay was performed. HepG2 cells were seeded into 96-well plates at a density of 2.5 × 10^4^ cells/well and incubated for 24 h. After removal of the culture medium, the cells were treated with various concentrations of IB and IBC in serum-reduced DMEM (1% FBS) for an additional 24 h. Subsequently, MTT solution was added to achieve a final concentration of 0.2 mg/mL, and the cells were incubated at 37 °C for 2 h in the dark. The supernatant was removed, and the resulting formazan crystals were dissolved in DMSO. The absorbance was measured at 540 nm using a microplate reader. All experiments were performed in triplicate. To account for potential interference of the extracts with MTT absorbance, extract-only blanks were included at each concentration and subtracted from the corresponding sample absorbance values.

### 2.12. Oil Red O Staining

HepG2 cells were seeded into 6-well plates at a density of 5 × 10^5^ cells/well and incubated for 24 h. To induce intracellular lipid accumulation, the cells were treated with 1 mM free fatty acids (FFAs), prepared as a 2:1 mixture of oleic acid (0.67 mM) and palmitic acid (0.33 mM) conjugated with 1% bovine serum albumin (BSA), for 24 h. The normal control (NC) group was treated with 1% BSA in the absence of FFAs, serving as the vehicle control. For co-treatment experiments, IB and IBC were added simultaneously with FFAs and incubated together for 24 h. After treatment, the cells were washed with phosphate-buffered saline (PBS) and fixed with 10% formalin for 1.5 h at room temperature. The fixed cells were rinsed with 60% isopropanol and air-dried. Subsequently, the cells were stained with Oil Red O solution for 15 min, followed by washing three times with distilled water. Stained lipid droplets were observed under a light microscope (DMI 6000, Leica, Wetzlar, Germany). For quantitative analysis, the intracellularly retained Oil Red O dye was eluted using 100% isopropanol, and the absorbance was measured at 510 nm using a spectrophotometer [30]. All experiments were performed in triplicate.

### 2.13. In Silico Network Analysis

To explore the potential mechanisms underlying the biological effects of *I. balsamina*, a bioinformatics-based network analysis was employed. Active compounds derived from the seeds of *Impatiens balsamina* (known as “Jixingzi” in traditional medicine) were obtained from the Traditional Chinese Medicine Systems Pharmacology Database (TCMSP). Compounds with oral bioavailability (OB) ≥ 30% and drug-likeness (DL) ≥ 0.18 were selected as candidate compounds. The corresponding target proteins of the selected compounds were collected from TCMSP. Overlapping targets associated with oxidative stress, lipid peroxidation, and lipid accumulation were selected for further network analysis. A compound–target interaction network was constructed using Cytoscape software (version 3.10.4). Protein–protein interaction (PPI) analysis of overlapping targets was performed using the STRING database, with a confidence score threshold of >0.7. Topological analysis of the PPI network was conducted using the NetworkAnalyzer plugin in Cytoscape, and hub genes were defined as the top-ranked nodes based on degree value. Functional enrichment analysis, including Kyoto Encyclopedia of Genes and Genomes (KEGG) pathway and Gene Ontology (GO) biological process analyses, was conducted using the Database for Annotation, Visualization and Integrated Discovery (DAVID). Pathways and processes with FDR < 0.05 were considered statistically significant. The top 20 enriched KEGG pathways and GO biological processes were selected based on statistical significance (*p*-value) and enrichment score for visualization. To identify potential upstream transcriptional regulators of the overlapping targets, transcription factor (TF) enrichment analysis was performed using ChEA3 (ChIP-X Enrichment Analysis Version 3; https://maayanlab.cloud/chea3 (accessed on 7 May 2026). The overlapping target gene list was submitted to ChEA3, and candidate TFs were prioritized based on the integrated mean rank score, which integrates evidence across multiple libraries including ENCODE ChIP-Seq, ReMap ChIP-Seq, Enrichr queries, GTEx coexpression, ARCHS4 coexpression, and literature-curated ChIP-Seq data.

### 2.14. Statistical Analysis

The experimental data were analyzed using GraphPad Prism (version 5.0; GraphPad Software, La Jolla, CA, USA). Standard curves were constructed using Microsoft Excel and PowerPoint (Microsoft, Redmond, WA, USA). Statistical differences between groups were evaluated by one-way analysis of variance (ANOVA) followed by Dunnett’s multiple comparison test. Results are presented as means ± standard deviations (SDs), and *p* < 0.05 was considered statistically significant. Unless otherwise stated, all experiments were performed in triplicate (n = 3), and results represent the mean of three independent experimental replicates. The DNA nicking assay was performed as a qualitative assessment and is presented as a representative gel image; this assay was not subjected to statistical analysis due to its qualitative nature.

## 3. Results

### 3.1. Callus Induction from Seeds of Impatiens balsamina L.

**Figure 1:**
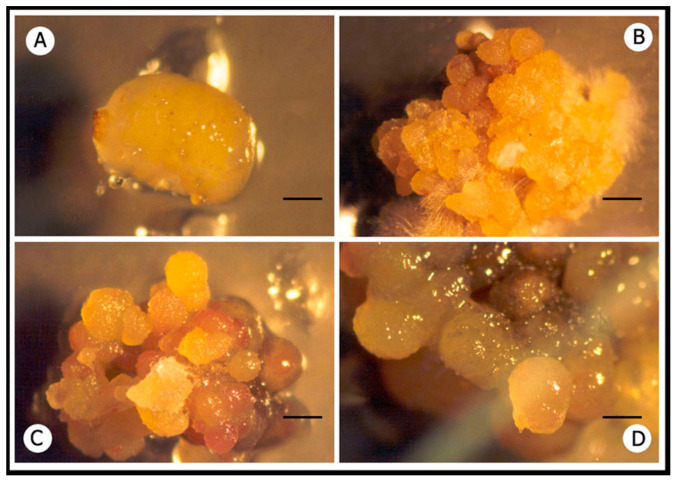
Callus induction from seeds of *I. balsamina*. (**A**) Seeds prior to culture. (**B**) Early-stage callus development at 4 weeks of culture. (**C**) Intermediate-stage callus at 8 weeks of culture. (**D**) Fully developed callus at 12 weeks of culture. All cultures were maintained on Murashige and Skoog (MS) medium supplemented with 1 mg/L 2,4-dichlorophenoxyacetic acid (2,4-D) in the dark at 28 °C. Magnification: 10× (**A**,**B**), 20× (**C**), 40× (**D**). Scale bars = 500 μm (**A**,**B**), 250 μm (**C**), and 125 μm (**D**).

Callus induction from seeds of *I. balsamina* was evaluated on Murashige and Skoog (MS) basal medium supplemented with various concentrations of 2,4-dichlorophenoxyacetic acid (2,4-D). The cultures were incubated in the dark for 3 months. In the absence of 2,4-D, the seeds germinated normally, and no callus formation was observed. Among the tested concentrations, the highest callus induction frequency was obtained at 1.0 mg/L 2,4-D, reaching 74.30% (Table 2). Callus induced at this concentration was well-developed, exhibiting a compact and glossy appearance without browning, as documented at 4, 8, and 12 weeks of culture (Figure 1).

### 3.2. Free Radical Scavenging Activities of IB and IBC

**Figure 2:**
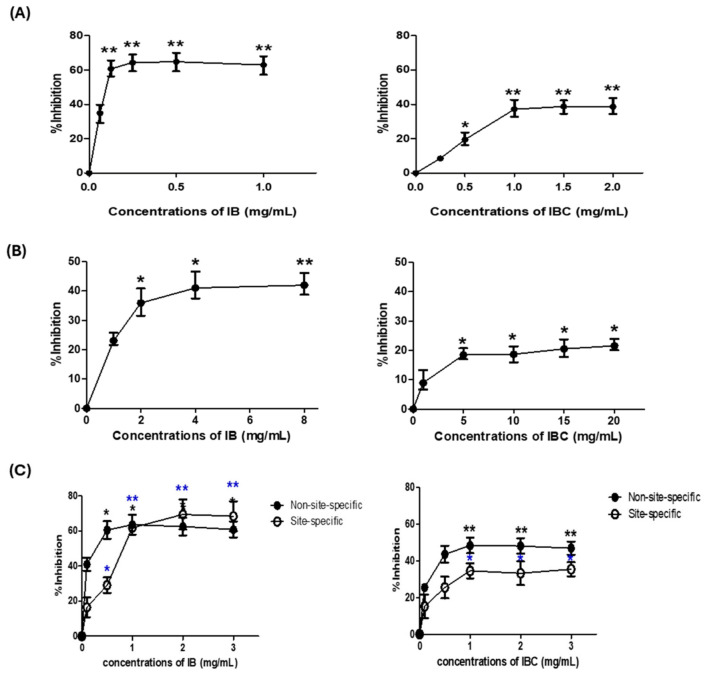
Free radical scavenging activities of *I. balsamina* seed extract (IB) and seed-derived callus extract (IBC). (**A**) DPPH radical scavenging activity expressed as percentage inhibition. The IC_50_ value of IB was 111.04 ± 8.69 μg/mL; the IC_50_ of ascorbic acid (positive control) was 3.78 μg/mL; the IC_50_ of IBC could not be determined within the tested concentration range. (**B**) Superoxide anion scavenging activity measured by inhibition of NBT reduction in the hypoxanthine–xanthine oxidase system. (**C**) Hydroxyl radical scavenging activity determined by the deoxyribose assay under non-site-specific (●) and site-specific (○) conditions. All values are presented as mean ± SD (n = 3). Black * *p* < 0.05, ** *p* < 0.01 indicate significance of non-site-specific conditions compared with the vehicle control. Blue * *p* < 0.05, ** *p* < 0.01 indicate significance of site-specific conditions compared with the vehicle control.

The free radical scavenging activities of IB and IBC were evaluated using DPPH, superoxide anion, and deoxyribose assays (Figure 2). Both IB and IBC exhibited concentration-dependent scavenging activity against DPPH radicals (Figure 2A). IB exhibited stronger activity, showing 60.7% inhibition at 0.125 mg/mL, whereas IBC reached 37.16% inhibition at 1 mg/mL. The IC_50_ value of IB was calculated as 111.04 ± 8.69 μg/mL, while that of IBC could not be determined within the tested concentration range due to insufficient maximal inhibition. For reference, the IC_50_ of ascorbic acid, used as a positive control, was 3.78 μg/mL. In the superoxide anion scavenging assay (Figure 2B), IB also showed higher activity than IBC, reaching 40.37% inhibition at ≥4 mg/mL. In contrast, IBC exhibited relatively weak activity, with a maximum inhibition of 18.50% at ≥5 mg/mL. Hydroxyl radical scavenging activity was further evaluated using the deoxyribose assay (Figure 2C). IB inhibited deoxyribose oxidation under both site-specific and non-site-specific conditions, with greater inhibition under non-site-specific conditions at lower concentrations (0.1–1.0 mg/mL) and under site-specific conditions at higher concentrations (2.0–3.0 mg/mL). In contrast, IBC consistently showed higher inhibition under non-site-specific conditions across all tested concentrations. Overall, IB exhibited stronger free radical scavenging activity than IBC across all assays.

### 3.3. Protective Effects of IB and IBC Against Oxidative Damage

**Figure 3:**
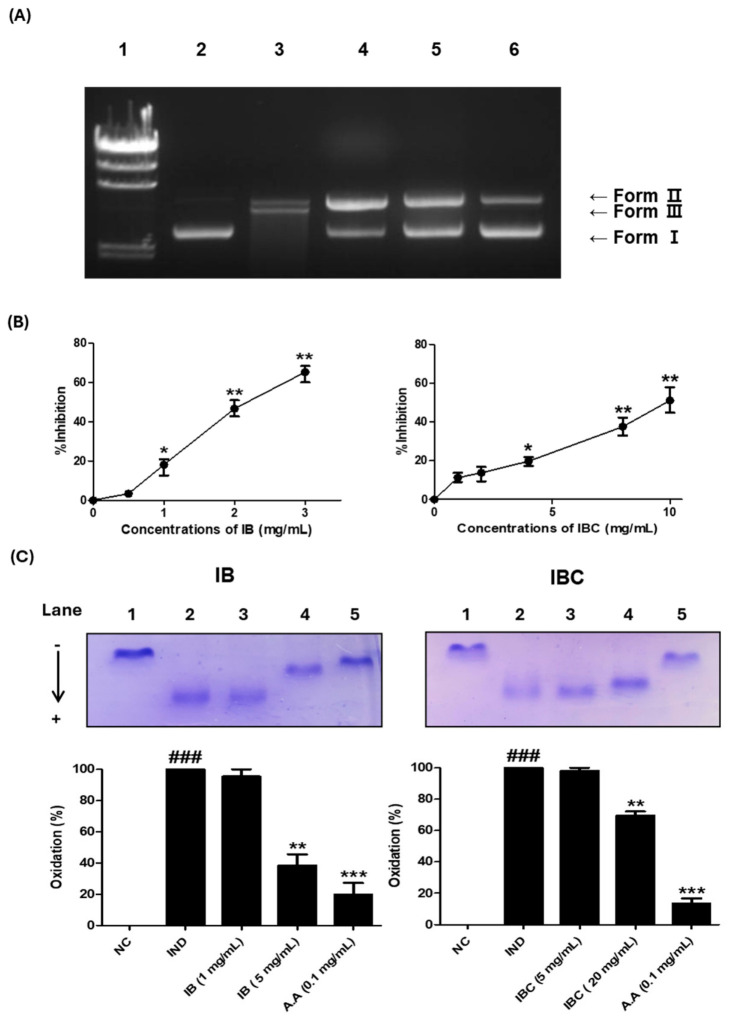
Protective effects of *I. balsamina* seed extract (IB) and seed-derived callus extract (IBC) against oxidative damage. (**A**) DNA nicking assay showing protection against hydroxyl radical-induced plasmid DNA damage. Lane 1: λ/Hind III DNA marker; Lane 2: native plasmid DNA; Lane 3: Fenton’s reagent-treated DNA; Lane 4: IB (5 mg/mL); Lane 5: IBC (20 mg/mL); Lane 6: catalase (2 U, positive control). Form I, supercoiled DNA; Form II, nicked circular DNA; Form III, linear DNA. (**B**) Inhibition of linoleic acid lipid peroxidation measured by the ammonium thiocyanate assay. The activities of IB and IBC are expressed as percentage inhibition. Values are presented as mean ± SD (n = 3). * *p* < 0.05, ** *p* < 0.01 compared with the vehicle control. (**C**) Inhibition of Cu^2+^-induced low-density lipoprotein (LDL) oxidation assessed by relative electrophoretic mobility (REM). Lane 1: native LDL (NC); Lane 2: Cu^2+^-induced oxidized LDL (IND); Lane 3: low concentration of extract (IB 1 mg/mL; IBC 5 mg/mL); Lane 4: high concentration of extract (IB 5 mg/mL; IBC 20 mg/mL); Lane 5: ascorbic acid (A.A, 0.1 mg/mL, positive control). NC, normal control; IND, Cu^2+^-induced LDL oxidation control. ### *p* < 0.001 compared with NC group; ** *p* < 0.01, *** *p* < 0.001 compared with IND group.

The protective effects of IB and IBC against oxidative damage to biomolecules were evaluated using DNA nicking, lipid peroxidation, and relative electrophoretic mobility (REM) assays (Figure 3). In the DNA nicking assay (Figure 3A), exposure of the pBR322 plasmid DNA to Fenton’s reagents resulted in the conversion of supercoiled DNA (Form I) to nicked circular (Form II) and linear (Form III) forms (lane 3). Treatment with IB (5 mg/mL) and IBC (20 mg/mL) preserved the supercoiled DNA (Form I) and reduced the formation of Forms II and III (lanes 4 and 5). The protective effects of IB and IBC were comparable to that of catalase (2 U, lane 6). In the lipid peroxidation assay (Figure 3B), IB inhibited Fe^3+^-induced linoleic acid oxidation in a concentration-dependent manner, reaching approximately 50% inhibition at 2–3 mg/mL. In contrast, IBC exhibited weaker activity and achieved similar inhibition only at higher concentrations (9–10 mg/mL). The protective effects of IB and IBC against Cu^2+^-induced oxidation of human low-density lipoprotein (LDL) were further evaluated using the REM assay (Figure 3C). Compared with native LDL (lane 1), CuSO_4_-treated LDL (lane 2) showed increased electrophoretic mobility toward the positive electrode. Treatment with IB (0.5 mg/mL) markedly reduced LDL migration, corresponding to 57% inhibition, which was comparable to ascorbic acid (0.1 mg/mL, 64%). In contrast, IBC exhibited only 16% inhibition even at 5 mg/mL. Overall, IB exhibited stronger protective effects against oxidative damage to DNA and lipids than IBC.

### 3.4. Total Phenolic, Flavonoid, and Tannin Contents

The total phenolic, flavonoid, and tannin contents of IB and IBC were determined (Table 3). The total phenolic content of IB was 10.72 ± 0.32 mg GAE/g extract, which was higher than that of IBC (4.72 ± 0.16 mg GAE/g extract). IB also exhibited a markedly higher total flavonoid content (103.27 ± 0.91 mg CE/g extract) compared with IBC (22.67 ± 2.27 mg CE/g extract). Similarly, the total tannin content was higher in IB (39.94 ± 1.28 mg TAE/g extract) than in IBC (11.57 ± 0.25 mg TAE/g extract). Overall, IB contained significantly higher levels of phenolic compounds, flavonoids, and tannins than IBC (*** *p* < 0.001).

### 3.5. Effects of IB and IBC on Cell Viability in HepG2 Cells

**Figure 4:**
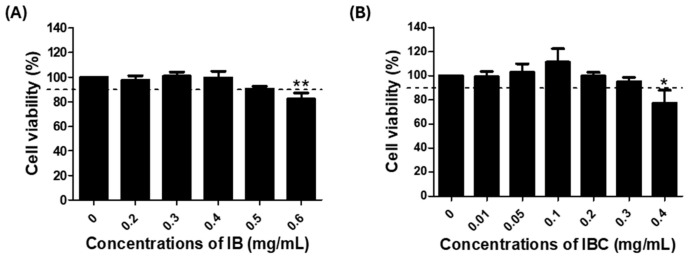
Effects of IB and IBC on cell viability in HepG2 cells. (**A**) Viability of HepG2 cells treated with IB (0–0.6 mg/mL). (**B**) Viability of HepG2 cells treated with IBC (0–0.4 mg/mL). Cells were treated for 24 h with the indicated concentrations. Cell viability is expressed as a percentage of the untreated control. The dashed line indicates 90% cell viability, used as the threshold for defining non-cytotoxic concentrations. Data are presented as mean ± SD (n = 3). * *p* < 0.05, ** *p* < 0.01 compared with the vehicle control.

Cell viability was evaluated using an MTT assay (Figure 4). IB exhibited no apparent cytotoxicity up to 0.5 mg/mL, with cell viability remaining above 90%. A statistically significant decrease in cell viability was observed at 0.6 mg/mL (** *p* < 0.01). Based on these results, IB concentrations of 0.1, 0.25, and 0.5 mg/mL were selected for subsequent experiments. IBC also showed no apparent cytotoxicity up to 0.3 mg/mL, whereas a statistically significant decrease in cell viability was observed at 0.4 mg/mL (* *p* < 0.05). Accordingly, IBC was used at concentrations of 0.05, 0.1, and 0.3 mg/mL for further experiments.

### 3.6. Effects of IB and IBC on Lipid Accumulation in HepG2 Cells

**Figure 5:**
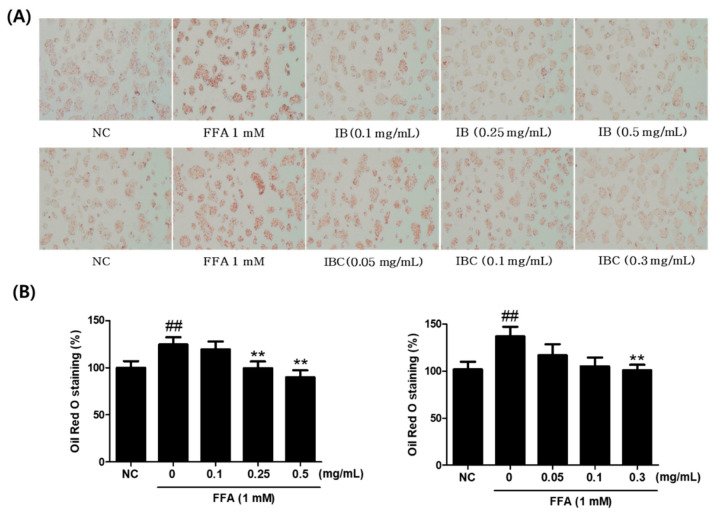
Effects of IB and IBC on lipid accumulation in HepG2 cells. (**A**) Representative Oil Red O staining images of HepG2 cells co-treated with 1 mM FFAs and IB or IBC for 24 h. The normal control (NC) group was treated with 1% BSA in the absence of FFAs. Images were captured at 40× magnification. (**B**) Quantification of intracellular lipid accumulation based on Oil Red O staining measured at 510 nm. Data are presented as mean ± SD (n = 3). ## *p* < 0.01 vs. NC group; ** *p* < 0.01 vs. FFA-treated group.

The effects of IB and IBC on intracellular lipid accumulation were evaluated in HepG2 cells exposed to free fatty acids (FFAs) for 24 h using Oil Red O staining (Figure 5). FFA treatment markedly increased lipid accumulation in HepG2 cells, as indicated by the formation of intracellular lipid droplets. IB treatment reduced lipid accumulation in a concentration-dependent manner. IBC also decreased lipid accumulation, although its inhibitory effect was weaker than that of IB. This was confirmed by the quantitative analysis of Oil Red O staining (Figure 5B). Overall, IB, and to a lesser extent, IBC, reduced Oil Red O staining in the FFA-induced HepG2 lipid accumulation model.

### 3.7. In Silico Network Analysis Reveals Potential Mechanisms Underlying the Antioxidant and Lipid Regulatory Effects

**Figure 6:**
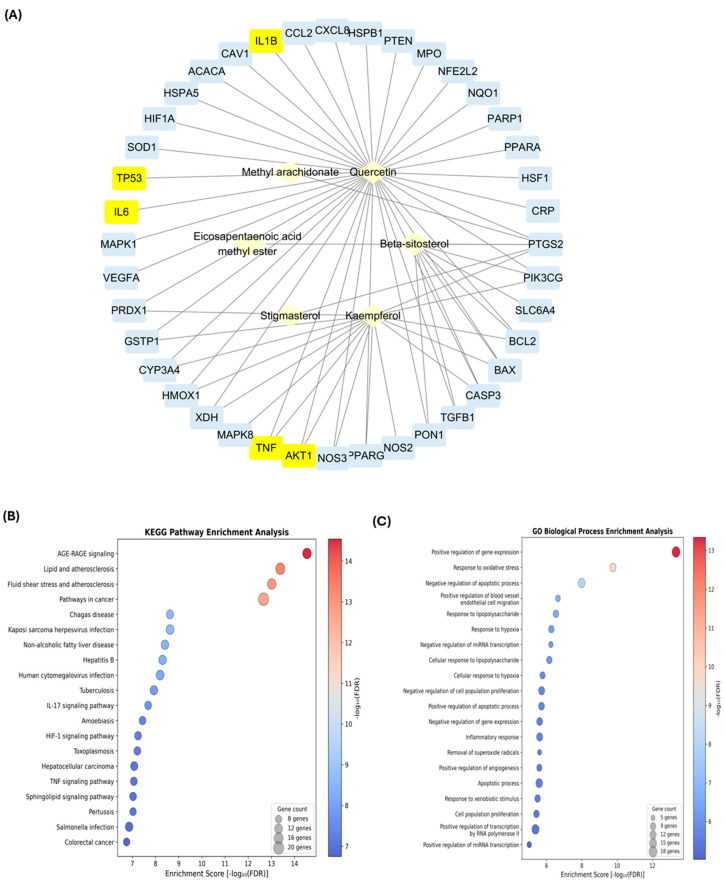
Integrated network-based analysis of bioactive compounds and potential mechanisms. (**A**) Compound–target interaction network. Yellow nodes represent bioactive compounds; blue nodes represent target proteins. Node size is proportional to the degree of connectivity. (**B**) KEGG pathway enrichment analysis bubble plot. (**C**) GO biological process enrichment analysis bubble plot. In (**B**,**C**), bubble size represents the number of enriched genes, and bubble color indicates the enrichment score based on −log_10_(FDR). The top 20 enriched terms are shown in order of statistical significance. All findings from this network analysis are hypothesis-generating and require future experimental validation.

To further elucidate the potential mechanisms underlying the biological activities of the seeds of *I. balsamina*, a bioinformatics-based network analysis was employed. Overlapping targets between compound-related targets and disease-associated targets linked to oxidative stress, lipid peroxidation, and lipid accumulation were identified and used to construct a compound–target interaction network (Figure 6A). The network analysis revealed that key compounds, including quercetin, kaempferol, and β-sitosterol, were connected to multiple targets, indicating their potential roles as major contributors to the observed biological activities. PPI network analysis further identified several hub genes, including *TNF*, *AKT1*, *TP53*, *IL6*, and *IL1B*, suggesting their central roles in regulating oxidative stress, inflammation, and lipid metabolism (Supplementary Figure S1). KEGG pathway enrichment analysis showed that the overlapping targets were significantly enriched in pathways associated with oxidative stress, inflammation, and lipid metabolism (Figure 6B). The most enriched pathways included AGE–RAGE signaling, lipid and atherosclerosis, fluid shear stress and atherosclerosis, non-alcoholic fatty liver disease (NAFLD), IL-17 signaling, TNF signaling, and HIF-1 signaling. Furthermore, GO biological process enrichment analysis indicated that the targets were mainly involved in oxidative stress response, inflammatory response, hypoxia response, lipopolysaccharide response, apoptosis, and regulation of cell proliferation (Figure 6C). Additional processes, including gene expression regulation and angiogenesis, were also enriched. Collectively, these results suggest that oxidative stress, inflammation, apoptosis, and lipid metabolism pathways may be relevant to the biological effects of *I. balsamina* seeds, although direct experimental validation remains to be conducted in future studies.

### 3.8. Transcription Factor Enrichment Analysis Identifies Upstream Regulators of Overlapping Targets

**Figure 7:**
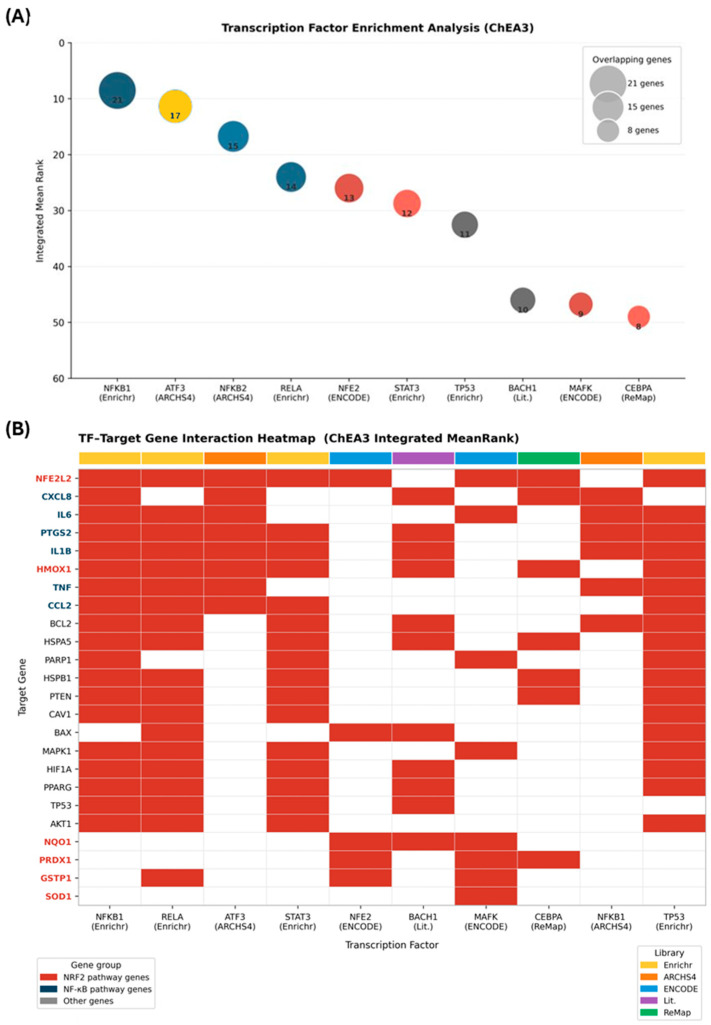
Transcription factor (TF) enrichment analysis of overlapping target genes using ChEA3. (**A**) Bubble plot showing the top 10 ranked TFs based on integrated mean rank score. The *Y*-axis represents the integrated mean rank score (lower values indicate higher ranking), and bubble size indicates the number of overlapping genes. Bubble colors indicate TF functional groups: blue, NF-κB family TFs (*NFKB1*, *NFKB2*, *RELA*); yellow, oxidative stress-inducible TF (*ATF3*); red, NRF2 pathway-related TFs (*NFE2*, *MAFK*) and hepatic metabolism-associated TFs (*STAT3*, *CEBPA*); gray, other TFs (*TP53*, *BACH1*). Note that NFE2 and MAFK are members of the CNC-bZIP family associated with antioxidant response element (ARE)-driven gene expression and are functionally distinguished from *NFE2L2* (NRF2) itself. (**B**) Heatmap showing TF–target gene interactions. Red gene labels indicate NRF2 pathway-associated genes; blue gene labels indicate NF-κB pathway-associated genes. The colored bar at the top represents the library source of each TF.

To identify potential upstream transcriptional regulators of the overlapping targets, TF enrichment analysis was performed using ChEA3. The integrated mean rank analysis revealed that *NFKB1* ranked highest with 21 overlapping genes, followed by *ATF3* (17 genes), *NFKB2* (15 genes), *RELA* (14 genes), *NFE2* (13 genes), *STAT3* (12 genes), *TP53* (11 genes), *BACH1* (10 genes), *MAFK* (9 genes), and *CEBPA* (8 genes) (Figure 7A). The TF–target gene interaction heatmap further illustrated the regulatory relationships between the top 10 TFs and the overlapping target genes (Figure 7B). Among the top-ranked TFs, *NFKB1*, *NFKB2*, and *RELA* are members of the NF-κB transcription factor family and were associated with the largest number of overlapping targets, including *IL6*, *IL1B*, *TNF*, *CXCL8*, *CCL2*, and *PTGS2*. *ATF3*, an oxidative stress-inducible transcription factor, was associated with overlapping targets including *HMOX1*, *NFE2L2*, *IL6*, *IL1B*, *TNF*, *CCL2*, *PTGS2*, and *CXCL8*, suggesting its potential role as a regulatory bridge between inflammatory and antioxidant responses. *NFE2* and *MAFK* showed preferential association with antioxidant-related target genes, including *NFE2L2*, *HMOX1*, *NQO1*, *PRDX1*, *GSTP1*, and *SOD1*. *CEBPA* was associated with 8 overlapping targets, including *NFE2L2*, *HMOX1*, *HSPA5*, *HSPB1*, *PTEN*, *CXCL8*, and *PRDX1*. Collectively, the top-ranked TFs were primarily associated with overlapping targets involved in inflammatory signaling, oxidative stress response, and hepatic lipid metabolism, consistent with the KEGG and GO enrichment results described above.

## 4. Discussion

Recent environmental changes, including habitat destruction and global warming, have accelerated the depletion of medicinal plant resources [31]. In this context, plant tissue culture technologies have emerged as promising strategies for the conservation of plant genetic resources and the sustainable production of bioactive compounds [32]. In particular, callus culture systems provide a controllable and scalable platform for the continuous production of plant-derived metabolites independent of environmental and seasonal variations [33].

In the present study, callus was successfully induced from seeds of *Impatiens balsamina* L. using different concentrations of 2,4-dichlorophenoxyacetic acid (2,4-D). The highest induction frequency was observed at 1.0 mg/L 2,4-D, where callus exhibited a healthy morphology without browning. These findings are consistent with previous reports suggesting that relatively low concentrations of auxins are optimal for callus induction, whereas higher concentrations may inhibit growth due to hormonal imbalance or cytotoxic effects [34].

The antioxidant activities of seed extract (IB) and seed-derived callus extract (IBC) were comparatively evaluated using multiple in vitro assays. IB consistently exhibited stronger antioxidant activity than IBC across radical scavenging, lipid peroxidation, and DNA protection assays, which is likely associated with the higher accumulation of phenolic compounds, flavonoids, and tannins in seeds. From a mechanistic perspective, IB exhibited both direct radical scavenging and metal chelation activities, whereas IBC primarily showed direct scavenging effects, suggesting a reduced diversity and abundance of antioxidant compounds in callus tissues. These observations are consistent with previous reports indicating that dedifferentiated plant cells often exhibit lower levels of secondary metabolite accumulation due to the absence of tissue-specific biosynthetic pathways. This may explain the relatively lower antioxidant activity observed in IBC compared to IB. Despite its relatively lower activity, IBC demonstrated measurable antioxidant effects across all assays, indicating that callus tissues retain the intrinsic biosynthetic capacity to produce bioactive compounds even in a dedifferentiated state. It should be noted, however, that this comparison was performed on an equal extract mass concentration basis. Since seeds and callus tissues differ substantially in water content, cellular organization, and metabolic composition, direct quantitative comparisons may not be fully biologically equivalent. Future studies employing normalization based on specific marker compounds or dry biomass-adjusted yields would provide a more rigorous basis for comparison.

In addition to the in vitro antioxidant assays, the effects of IB and IBC on lipid accumulation were evaluated using an FFA-induced HepG2 cell model. FFA exposure increased intracellular lipid droplet formation, whereas both IB and IBC reduced lipid accumulation in a concentration-dependent manner. Oxidative stress plays a critical role in lipid metabolism and the development of hepatic lipid accumulation [35,36]. However, it should be noted that these observations are based solely on in vitro experiments, and further in vivo studies are needed to substantiate the physiological relevance of these findings. Furthermore, the Oil Red O staining assay used in this study is a well-established semi-quantitative method for screening intracellular lipid accumulation in hepatocyte models. While additional endpoints such as triglyceride quantification or lipid metabolism gene expression analysis would further substantiate these findings, the present study was designed as a preliminary comparative investigation. Future studies will incorporate more detailed mechanistic analyses to clarify the relationship between antioxidant capacity and lipid regulatory effects. Notably, the stronger inhibitory effect of IB is consistent with its higher antioxidant capacity and phytochemical content.

To explore potential mechanisms that may underlie the observed biological activities, a bioinformatics-based network analysis was employed as a hypothesis-generating approach. The in silico network analysis provides a hypothesis-generating framework for understanding potential mechanisms underlying the biological effects of *I. balsamina* seeds. It should be noted that this analysis was based on literature-derived compounds, and detailed phytochemical profiling of the actual IB and IBC extracts was not performed in the present study. Therefore, the identified pathways and transcription factors should be interpreted as hypotheses to be tested in future studies rather than mechanistically validated conclusions. With this caveat in mind, among the identified compounds, flavonoids such as quercetin and kaempferol, along with the phytosterol β-sitosterol, were connected to multiple targets, suggesting their potential roles as key bioactive constituents. Kaempferol is known to exert antioxidant and anti-inflammatory effects through the modulation of redox-sensitive signaling pathways, including NF-κB, MAPK, and PI3K/AKT [37,38]. β-Sitosterol is also known to regulate lipid metabolism and attenuate lipid accumulation by modulating cholesterol homeostasis and inflammatory signaling pathways [39,40]. Consistent with these properties, enrichment analysis highlighted pathways such as AGE–RAGE, TNF, and HIF-1 signaling, as well as biological processes related to oxidative stress, apoptosis, and lipid metabolism [41,42]. These findings suggest that oxidative stress, inflammation, and lipid metabolism pathways may be relevant to the biological activities of *I. balsamina* seeds, based on in silico hypothesis-generating analysis.

TF enrichment analysis further identified *NFKB1*, *NFKB2*, and *RELA* as the top-ranked upstream regulators of the overlapping targets, collectively governing the transcriptional regulation of pro-inflammatory mediators including *IL6*, *IL1B*, *TNF*, and *PTGS2*. These results are consistent with previous reports demonstrating that quercetin and kaempferol exert anti-inflammatory effects through the suppression of NF-κB transcriptional activity [43]. *ATF3*, ranked second among the identified TFs, is an oxidative stress-inducible transcription factor that has been reported to modulate both NF-κB signaling and antioxidant gene expression, suggesting its potential role as a regulatory bridge between inflammatory and antioxidant responses. Furthermore, *NFE2* and *MAFK*, members of the CNC-bZIP family, were preferentially associated with ARE-driven antioxidant target genes such as *HMOX1*, *NQO1*, and *GSTP1*, providing in silico support for the involvement of NRF2-associated transcriptional regulation in the antioxidant effects of *I. balsamina*. Notably, *CEBPA*, a transcription factor involved in hepatic lipid metabolism, was also identified among the top-ranked regulators. Given its established role in regulating fatty acid metabolism and lipid homeostasis in hepatocytes, the identification of *CEBPA* suggests a potential transcriptional basis for the lipid accumulation-inhibitory effects observed in FFA-treated HepG2 cells, further corroborating the link between antioxidant activity and lipid regulatory mechanisms. Consistent with these findings, previous transcriptomic studies have reported that *TNF*, *AKT1*, and *IL6* are significantly upregulated in hepatic tissues of NAFLD patients compared with healthy controls [44], further supporting the disease relevance of the hub genes identified in the present network analysis.

From a biotechnological perspective, the significance of callus culture lies not in its immediate superiority over natural plant tissues, but in its potential for optimization and industrial application [45]. Unlike seeds, callus cultures can be maintained under controlled conditions and are amenable to enhancement strategies such as elicitor treatment, precursor feeding, metabolic engineering, and bioreactor-based scale-up [46]. Although the antioxidant activity of IBC was lower than that of IB, its baseline activity suggests that callus-derived materials may warrant further investigation as a potential source of bioactive compounds, pending systematic optimization of culture conditions and metabolite productivity. Several limitations of the present study should be acknowledged. First, all experiments were conducted using in vitro systems, and the observed biological effects have not been validated in animal or clinical models. Therefore, broader conclusions regarding the prevention or treatment of metabolic diseases such as NAFLD remain speculative and require further in vivo investigation. In addition, the lipid accumulation assessment was based on Oil Red O staining as a semi-quantitative screening method, and additional biochemical endpoints such as intracellular triglyceride quantification or cytotoxicity assessment under co-treatment conditions were not included in the present study. These aspects should be addressed in future investigations to provide more comprehensive characterization of the lipid regulatory effects. Second, the callus induction system was applied at a basic level using only 2,4-D as the growth regulator, without further optimization through elicitor treatment, alternative plant growth regulators, light conditions, or bioreactor-based scale-up approaches. Accordingly, the potential of seed-derived callus as a production platform remains to be further validated through systematic optimization studies. Third, the comparison between IB and IBC was based on extract mass concentration; however, seeds and callus tissues differ substantially in water content, cellular organization, and metabolic composition. Whether these comparisons are fully biologically equivalent, or whether normalization based on specific marker compounds would provide a more accurate comparison, warrants further investigation. Finally, the in silico network analysis was based on literature-derived compounds associated with *I. balsamina* seeds, and detailed LC-MS/MS characterization of the actual IB and IBC extracts was not performed. Therefore, the connection between the computational predictions and the experimental results remains indirect, and the identified pathways and transcription factors should be interpreted as hypothesis-generating rather than mechanistically validated findings. Taken together, these findings suggest that while seeds of *I. balsamina* exhibit superior antioxidant capacity, seed-derived callus may represent a conceptually promising starting point for the development of a scalable production system, pending further experimental optimization.

## 5. Conclusions

In this study, the antioxidant activities of seed extract (IB) and seed-derived callus extract (IBC) of *Impatiens balsamina* L. were comparatively evaluated using multiple in vitro assays. Both IB and IBC exhibited significant antioxidant effects, including free radical scavenging, inhibition of lipid peroxidation, and protection against oxidative DNA damage, with IB showing higher activity than IBC, likely due to its greater phytochemical content. In addition, both extracts attenuated FFA-induced lipid accumulation in HepG2 cells, suggesting a possible association between antioxidant activity and lipid regulation. Despite its relatively lower activity, IBC retained measurable bioactivity, indicating that callus tissues maintain the capacity to produce functional metabolites. Furthermore, in silico network and transcription factor enrichment analyses generated hypotheses suggesting that NF-κB and NRF2-associated transcriptional networks may be relevant to these biological effects; however, these predictions were not directly validated experimentally and should be tested in future mechanistic studies. Overall, these findings suggest that while seeds of *I. balsamina* possess superior antioxidant potency, seed-derived callus may serve as a conceptually promising starting point for the future development of a sustainable production system, with its practical utility to be demonstrated through further systematic optimization.

## Figures and Tables

**Table 1:** Composition of culture medium used for callus induction of *I. balsamina*.

Compound	MS Medium(mg/L)
NH_4_NO_3_	1650.0
KNO_3_	1900.0
CaCl_2_·2H_2_O	440.0
MgSO_4_·7H_2_O	370.0
KH_2_PO_4_	170.0
MnSO_4_·4H_2_O	22.3
ZnSO_4_·7H_2_O	8.6
H_3_BO_3_	6.2
KI	0.83
NaMoO_4_·2H_2_O	0.25
CuSO_4_·5H_2_O	0.025
CoCl_2_·6H_2_O	0.025
Na_2_EDTA	37.3
FeSO_4_·7H_2_O	27.8
Thiamine·HCl	0.1
Pyridoxine·HCl	0.5
Nicotinic acid	0.5
Inositol (g/L)	0.1
Caseinhydrolysate (g/L)	1.0
Phytagel (g/L)	4.0
Sucrose (g/L)	30.0
pH	5.7

**Table 2:** Effect of 2,4-dichlorophenoxyacetic acid (2,4-D) on callus induction from seeds of *I. balsamina*. Callus induction frequency (%) was calculated based on the number of seeds that formed callus after 3 months of culture. Callus morphology was qualitatively assessed based on appearance, size, and browning.

2,4-D (mg/L)	Callus Induction Rate (%)	Callus Morphology
0	0	-
0.5	69.20	+
1.0	74.30	+++
2	52	++
5	37	+

Callus morphology was assessed based on visual inspection of callus size, compactness, color, and browning. -, no callus formation observed; +, small and loosely organized callus with slight browning; ++, moderate callus formation with partial browning; +++, well-developed, compact, and glossy callus without browning. Callus induction frequency was determined from a single experimental run with 25 explants per treatment. Statistical analysis was not performed due to the single-experiment design.

**Table 3:** Total phenolic, tannin, and flavonoid contents of *I. balsamina* seed extract (IB) and seed-derived callus extract (IBC). Results are expressed as mg gallic acid equivalents (GAE)/g extract, mg catechin equivalents (CE)/g extract, and mg tannic acid equivalents (TAE)/g extract, respectively. Values are presented as mean ± SD (n = 3). *** *p* < 0.001, significantly different between IB and IBC by unpaired *t*-test.

Sample	Total Phenolic (mg GAE/g extract)	Total Tanin (mg TAE/g Extract)	Total Flavonoid (mg CE/g Extract)
*I. balsamina* (IB)	10.72 ± 0.32	39.94 ± 1.28	103.27 ± 0.91
*I. balsamina* callus (IBC)	4.72 ± 0.16 ***	11.57 ± 0.25 ***	22.67 ± 2.27 ***

## Data Availability

The original contributions presented in this study are included in the article/Supplementary Materials. Further inquiries can be directed to the corresponding author.

## Supplementary Materials

The following supporting information can be downloaded at: https://www.mdpi.com/article/10.3390/plants15111716/s1, Supplementary Figure S1. Protein–protein interaction (PPI) network of overlapping targets associated with oxidative stress, lipid peroxidation, and lipid accumulation. The PPI network was constructed using the STRING database with a confidence score threshold of >0.7. Hub genes were identified based on degree values, and the top-ranked genes included *TNF*, *AKT1*, *TP53*, *IL6*, and *IL1B*.

## Abbreviations

2,4-D	2,4-Dichlorophenoxyacetic acid
CE	Catechin equivalent
DPPH	2,2-Diphenyl-1-picrylhydrazyl
EDTA	Ethylenediaminetetraacetic acid
FBS	Fetal bovine serum
FFA	Free fatty acid
GAE	Gallic acid equivalent
HepG2	Human hepatocellular carcinoma cells
H_2_O_2_	Hydrogen peroxide
IB	*Impatiens balsamina* L. seed extract
IBC	*Impatiens balsamina* L. callus extract
LDL	Low-density lipoprotein
MS	Murashige and Skoog medium
REM	Relative electrophoretic mobility
ROS	Reactive oxygen species
TAE	Tannic acid equivalent
