# Supplementary material for: Antioxidant Activities and Lipid Accumulation-Inhibitory Effects of Seed and Callus Extracts of Impatiens balsamina L
Source: Plants (Basel). 2026 Jun 1;15(11):1716. doi: 10.3390/plants15111716 (PMC13259232; doi:10.3390/plants15111716)
Supplement: Supplementary file 1 [file plants-15-01716-s001.zip › plants-4340436-supplementary.pdf]

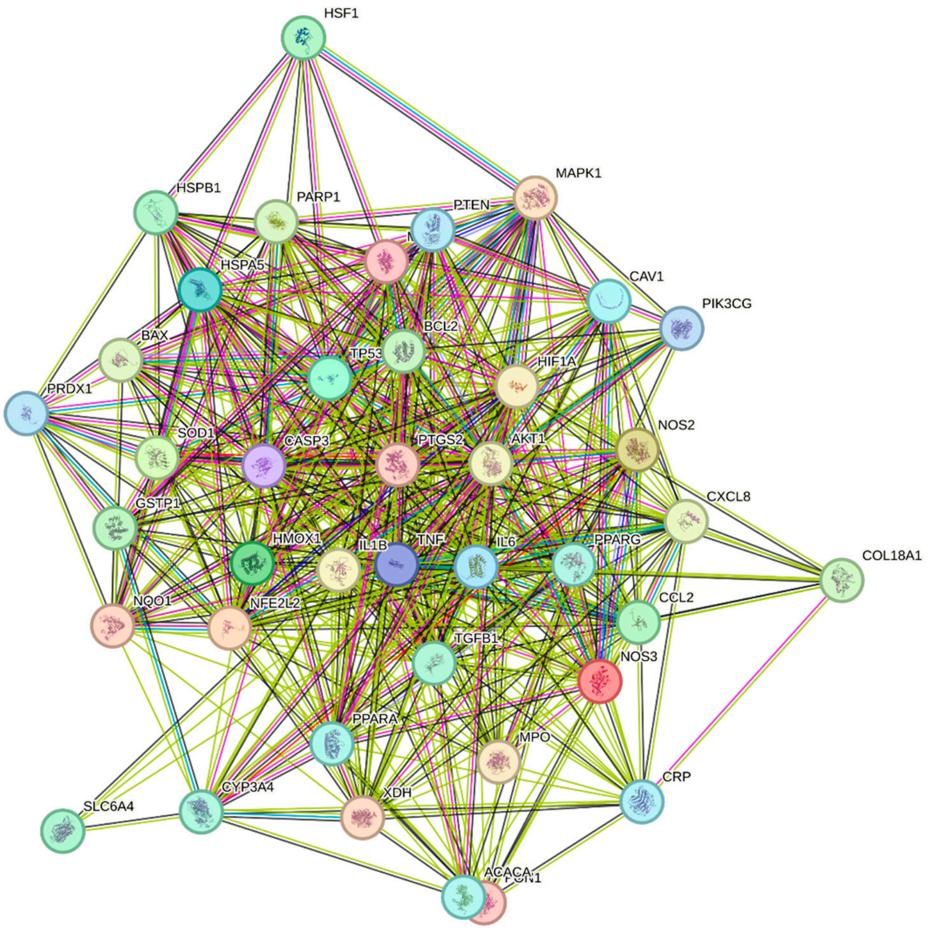

Supplementary Figure S1. Protein–protein interaction (PPI) network of overlapping targets associated with oxidative stress, lipid peroxidation, and lipid accumulation. The PPI network was constructed using the STRING database with a confidence score threshold of > 0.7. Hub genes were identified based on degree values, and the top-ranked genes included TNF, AKT1, TP53, IL6, and IL1B.
